# Human Testicular Tissue Digestion, Testicular Cell Selection, and Downstream Characterization for Reproductive Purposes: A Scoping Review

**DOI:** 10.3390/ijms262010150

**Published:** 2025-10-18

**Authors:** Sven De Windt, Neguine Nekounazar Azad, Christine Wyns

**Affiliations:** 1 Pôle de Recherche en Physiopathologie de la Reproduction, Institut de Recherche Expérimentale et Clinique (IREC), Université Catholique de Louvain, 1200 Brussels, Belgium; sven.dewindt@uclouvain.be; 2 Department of Gynecology-Andrology, Cliniques Universitaires Saint-Luc, 1200 Brussels, Belgium

**Keywords:** immature testicular tissue (ITT), childhood cancer, enzymatic digestion, mechanical dissociation, spermatogonial stem cells (SSCs), fertility preservation, enrichment, cell isolation, flow cytometry, density gradient

## Abstract

Fertility preservation and restoration using cryo-banked prepubertal testicular tissue is a pivotal part of the childhood hematological cancer care pathway. Estimations indicate that one in 900–1400 young adults is a childhood cancer survivor, underlying the urge to develop fertility restoration protocols as some of the patients have reached the age to father their own genetic child. While it has been reported that 39% of patients present cancer cells in their testes, no efficient decontamination technique has been identified to circumvent cancer reintroduction after autologous testicular cell transplantation. Obtaining single-cell suspensions and selecting only testicular cells might be an option. In this review, mechanical dissociation/enzymatic digestion protocols applied to human testicular tissue, as well as selection and enrichment strategies, and their outcome will be presented and discussed. While the literature revealed a plethora of mechanical dissociation/enzymatic digestion protocols, testicular tissue characteristics are often missing, precluding the comparison of protocols and their outcomes. Downstream selection and enrichment strategies showed promising results with flow cytometry reaching fractions with the highest purity. Future studies should focus on investigating digestion outcomes to elucidate potential influences on both the cell type-specific viability and the cell-to-cell interactions necessary for cell proliferation and differentiation of selected or enriched testicular cell types. Such research outputs will then also be crucial for further progress in in vitro spermatogenesis from testicular cell suspensions as another option for patients that banked testicular tissue at the time of a hematological cancer.

## 1. Introduction

Thanks to improvements in cancer therapies over the last years, pediatric cancer survival rates have increased to more than 80% [1]. Estimations predict one in 900 to 1400 young adults to be a childhood cancer survivor [2]. Unfortunately, these cancer therapies may lead to impaired fertility in adulthood. Extending indications of drugs affecting fertility to benign disease increases even more the population concerned by this matter. Indeed, while it was shown that the majority of patients exposed to gonadotoxic therapies only experience transient fertility impairment and spermatogenesis recovery 1–5 years post-treatment [3], still 42.2% to 66.4% of the patients has germ cell (GC) failure with resulting infertility whether being prepubertal, peripubertal or postpubertal at the time of treatment [4,5,6,7,8]. For prepubertal boys, unlike adolescent boys and adult men who can benefit from conventional semen freezing, cryopreservation of immature testicular tissue (ITT) containing spermatogonial stem cells (SSCs) is the only experimental fertility preservation option which is ethically accepted to offer these patients a chance of fathering their own genetic child [9,10,11,12,13]. Survey data show that this approach is increasingly proposed worldwide [14,15].

Despite the urge, as some of the patients have reached the age to conceive [16], still no fertilizing spermatozoa have been obtained from ITT. Several methods have been investigated to reproduce spermatogenesis with cryopreserved ITT, i.e., in vitro maturation (IVM) and transplantation of single-cell suspensions or ITT fragments [13]. While autotransplantation appears to be the most promising technique with the generation of mature sperm and offspring in monkeys [17], a major concern is the possibility of cancer cell contamination of cryo-stored ITT [18,19], especially in the case of hematological malignancies showing a mean contamination rate in pre- and peripubertal boys between 37 and 39% [18,19], carrying thereby the risk of causing disease relapse if autotransplanted [20]. Indeed, it was shown that as few as 20 leukemic cells are sufficient to reinduce cancer in rats [21]. Therefore, autotransplantation of human ITT grafts is currently excluded when there is a risk of cancer cell contamination of the tissue. Xenotransplantation of human ITT and retrieval of spermatozoa to be used for in vitro fertilization with intracytoplasmic sperm injection (IVF-ICSI) would circumvent this risk but is clinically unaccepted due to xeno-microbiological agents [22]. IVM of human ITT or testicular cell suspensions aimed at differentiating SSCs in spermatozoa to be further used for IVF-ICSI would be the optimal solution to overcome the risk of cancer cell contamination and disease relapse. However, it is still at the research stage and limited to the generation of haploid spermatids from IVM of tissue fragments [23] and of cells phenotypically resembling spermatozoa after 3D-testicular cell culture [24]. Autotransplantation of properly selected spermatogonia including SSCs into the original testicular niche could be more promising based on the development of preimplantation embryos in non-human primates [25]. In addition, very recently a protocol for the autotransplantation of testicular cells in humans has been reported, pointing to the fast evolution in available methods [26] and the urgent clinical need of proper cell selection techniques. Alternatively, in vitro cancer-free-organoid formation in which selected testicular cells will colonize into an extracellular matrix (ECM)-based hydrogel/scaffold prior to transplantation could be considered [27,28]. Therefore, cell selection seems a relevant technique to obtain cancer-free single-cell suspension and guarantee safe autotransplantation. Currently, clinical implementation of the various cell selection techniques is hampered by inefficient cancer cell decontamination, risk of toxic components, requirements for post-sorting applications, low cell recovery, and limited standardization (Table 1).

**Figure 1:**
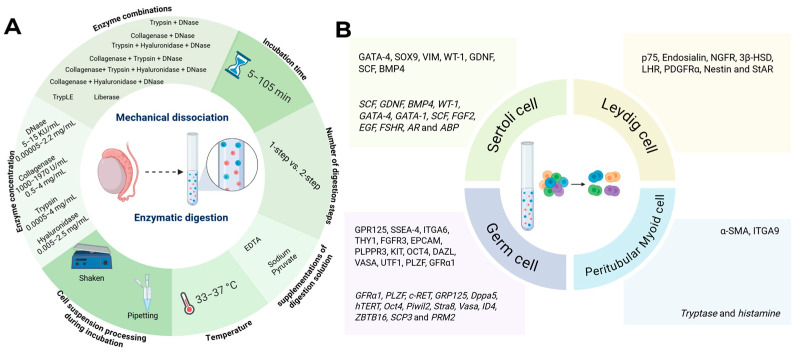
Graphical overview of the methods and markers used in the included papers. (**A**) Mechanical dissociation/enzymatic digestion variables including incubation time, supplementations of digestion solution, temperature, enzyme combinations and concentrations, number of digestion steps and cell suspension processing during incubation. Variables are presented in ranges if applicable. (**B**) Markers for testicular cell positivity, at protein and gene-level (italics) used during cell selection and downstream verification. Created in BioRender, https://BioRender.com/2c3f9cg.

**Figure 2:**
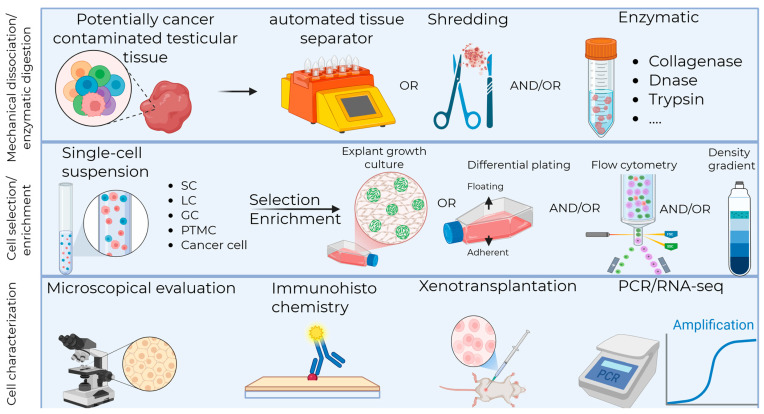
Testicular tissue mechanical dissociation/enzymatic digestion protocols and cell selection/enrichment strategies for downstream fertility restoration strategies. SC, Sertoli cell; LC, Leydig cell; GC, germ cell; PTMC, Peritubular Myoid cell; PCR, polymerase chain reaction; RNA-seq, ribonucleic acid sequencing. Created in BioRender, https://BioRender.com/50ykhfj.

To obtain single-cell suspensions, demonstrating high viability while maintaining cellular diversity and preserving rare subsets of testicular cells, a plethora of methods and markers are currently being used by different teams worldwide (Figure 1). Notably, detailed information on tissue digestion protocols with reporting of cell yields and numbers of viable cells is scarce, challenging protocol standardization, study comparison, and the interpretation of results. This systematic review aims to summarize the current literature on testicular tissue dissociation/digestion protocols; the cell selection and enrichment strategies, including the post-selection characterization of the major testicular subtypes by morphology and cell-specific markers at both the gene and protein levels (Figure 2).

## 2. Materials and Methods

### 2.1. Study Design

This is a scoping review written via the PRISMA guidelines and published protocol in the Open Science Framework network (https://osf.io/82mp4/ (accessed on 9 September 2025)) [31].

### 2.2. Literature Search

Medline and its electronic database PubMed were used to search articles related to the topic of the review, using the queries listed in Table 2. All research publications published until 31 May 2025 were included. Forward and backward snowballing was applied to identify extra articles of interest in references and citations, respectively.

### 2.3. Eligibility Criteria

Original research papers, written in English on experiments conducted on human testicular tissue by using the built-in search engine criterium were included. All reports containing testicular tissue dissociation, enzymatic digestion, selection or enrichment techniques, regardless of the final aim of the papers, were considered for inclusion. Exclusion criteria were articles published in languages other than English, reviews, guidelines, books, protocols without reporting results, research articles, and scientific video protocols. Papers reporting only outcomes on the selection/enrichment of differentiated GCs, being already standard of care in medically assisted reproduction, were not included. Any additional references found in original articles or review papers that were found relevant and missing from the primary search were added.

### 2.4. Study Selection

**Figure 3:**
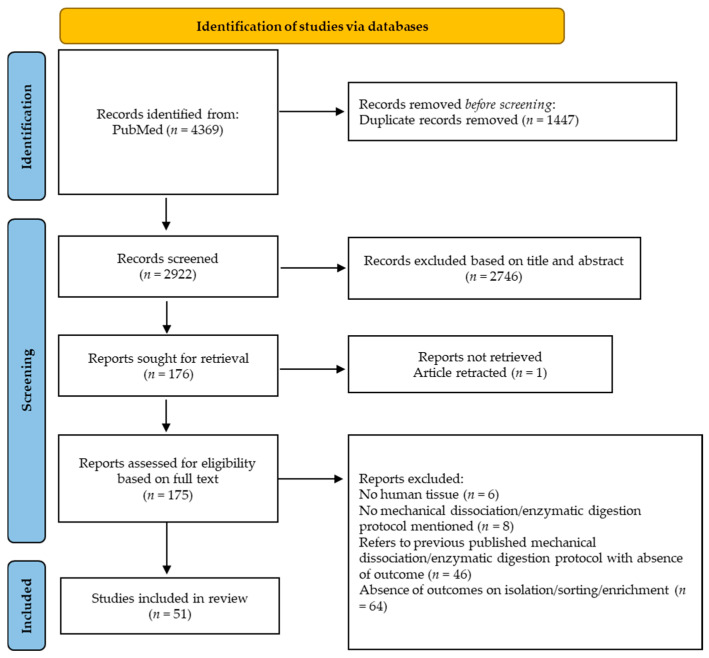
General flowchart of article selection process.

Title and abstract screening were performed by S.D.W. and N.N.A. in a blinded, independent, and separate way. These two searches were compared and discussed between S.D.W. and N.N.A. and in all cases, consensus was reached. The PRISMA flowchart illustrating the selection process of references is illustrated in Figure 3.

### 2.5. Data Extraction and Interpretation

Full text screening was performed by S.D.W. and N.N.A. in a blinded and independent manner. EndNote (V.21.0.1.) and Excel (Microsoft, V2311 Build 16.0.17029.20177) were used for the full text screening process. The results were, again, discussed among S.D.W. and N.N.A., and in all cases, consensus was reached. The data that was extracted consisted of the mechanical dissociation/enzymatic digestion protocols and corresponding cellular yield and/or viability. For cell selection, the data extracted consisted of the selection strategy, markers, and purity outcomes with corresponding verification techniques. When cell viability or yield were not reported, those outcomes were calculated provided viable and death cell quantification numbers and weight of digested testicular tissue were mentioned.

## 3. Results and Discussion

In total, 4369 articles were identified from PubMed. After the removal of duplicate records, 2922 were screened based on title and abstract. A total of 51 studies fulfilled the eligibility criteria and were included in this scoping review. The papers are classified based on dissociation/digestion protocol applied and the cell type to sort/enrich. For each paper, the first author, year of publication, age of the donor, medical diagnosis/tissue source/anatomopathology, number of donors, tissue weight/size, storage of testicular tissue, dissociation/digestion protocol, and/or outcomes are mentioned.

### 3.1. Testicular Tissue Dissociation/Digestion to Obtain Single-Cell Suspensions

Different protocols of dissociation/digestion have been reported. While most publications used enzymatic digestion to obtain single-cell suspensions, some, mostly older publications, used mechanical dissociation. Mechanical dissociation can be described as dissociating tissue fragments by applying physical force ranging from simple shredding [32] to (automated) tissue separators [33,34,35]. Enzymatic digestion uses digestive enzymes to break down the ECM to obtain isolated seminiferous tubules followed by cleavage of cell–cell junctions, often carried out in multi-step protocols [36,37]. Both strategies have the objective to create a single-cell suspension with high viable cell numbers, minimal cell debris or aggregates, while simultaneously preserving cellular integrity including membrane receptors for downstream selection applications and cell functioning [36]. Single-cell suspensions can then either be used as a complete cellular mix or as suspensions of selected or enriched specific cell types.

A complete overview of reported mechanical dissociation/enzymatic digestion protocols with their main outcomes regarding cell yield and viability is provided in Table 3.

As numerous protocols are reported in publications that do not aim to focus on single-cell suspension outcomes, dissociation or digestion protocols are often vaguely described without specific information on start material characteristics and data on total or cell-type-specific viability and yield.

We identified only one publication in which mechanical dissociation and enzymatic digestion were compared while reporting specific outcomes such as overall cell yield, viability, and influence on testicular cell types [33]. An automated tissue separator called “the Medimachine” to mechanically dissociate fresh human adult testicular tissue was compared to a commonly applied two-step enzymatic digestion protocol using collagenase I, followed by trypsin in combination with DNase. Although the Medimachine approach provided considerable advantages regarding lab time and costs, the overall viability was higher for enzymatic digestion compared to mechanical dissociation, resulting in 76% and 38% viability when using fresh testicular tissue, and 74% and 16% when starting with cryopreserved testicular tissue, respectively. Interestingly, investigating the proportion of undifferentiated GCs demonstrated an increase in SALL4+ undifferentiated GCs when cryopreserved tissue and fresh tissue were mechanical dissociated compared to enzymatic digested with 26.92 ± 6.52% versus 27.33 ± 9.49% and 7.95 ± 4.38% versus 19.58 ± 7.88 SALL4+ cells, respectively [33].

Only few studies used mechanical dissociation to obtain single-cell suspensions from testicular tissue [33,35]. Kurpisz et al., 1988 [35], used a previously published mechanical isolation protocol [38] based on multiple chilled razor blades to dissociate and mince adult tissue. While the authors reported the suspension to contain 20–25% of Sertoli cells (SCs), they observed extensive SC damage. Somatic cells’ vulnerability to mechanical dissociation was also observed by Schneider et al., 2015 [33], using the “Medimachine” automated tissue separator as significant lower vimentin (VIM) expression was observed after mechanical dissociation compared to enzymatic digestion.

Enzymatic digestion was applied following a plethora of protocols including various types and concentrations of enzymes, one-step vs. multistep strategies, different incubation times and conditions considering the maturation stage of the tissue (fetal, pre- or peripubertal, adult), some other tissue specificities (diseased or healthy, less or more fibrotic) and the downstream applications, e.g., IVM, auto-transplantation of single-cell suspension. Different subtypes of the same enzyme also influence outcomes. Collagenase IV, a protease secreted by SCs [39] has less tryptic activity leading to a less harmful effect on membrane receptors compared to the less pure, more crude, collagenase I. Although a direct comparison on human ITT is still awaited, a study conducted on rat hepatocytes already identified collagenase IV to better preserve the activity of specific membrane receptors [40]. The superiority of collagenase IV (versus collagenase I) was also shown when used to extract round spermatids and spermatozoa from adult testicular tissue leading to a higher proportion of vital cells and less debris [41]. Despite these observations, collagenase I was used in recent studies, but outcomes of the enzymatic digestion were unfortunately not available [42].

#### 3.1.1. Testicular Tissue Characteristics Influencing Testicular Dissociation/Digestion Outcomes

One of the most important characteristics of testicular tissue to be dissociated or digested is its prior cryopreservation or not, as cryopreservation can be seen as a first step of tissue dissociation. Cryopreservation can lead to membrane damage [43] especially for ITT proven to be twice as susceptible to this damage compared to adult tissue [43,44]. Indeed, significant differences between fresh and frozen/thawed testicular biopsies have been found both after mechanical dissociation and enzymatic digestion [33,45,46]. Zheng et al., 2014 [45], obtained significant lower overall cell yield when frozen/thawed tissue was enzymatically digested, though it is of note that all fresh tissue fragments were derived from the same patient, limiting the interpretation of these results. In line with their observation, lower viability (74.0% viability and 1.59 × 10^4^ viable cells/mg) was also observed by Pacchiarotti et al., 2013 [46], after enzymatic digestion of frozen/thawed versus fresh tissue (90.1% viability and 4.25 × 10^4^ cells/mg) from adult sexual reassignment biopsies. However, in his comparative study, Schneider et al., 2015 [33], obtained 16% and 38% living cells when mechanically dissociating adult human frozen/thawed and fresh testicular fragments, respectively, while no significant difference was observed for enzymatic digestion (74% and 76% viability, respectively, for adult human frozen/thawed and fresh testicular tissue).

#### 3.1.2. Protocol-Related Factors Influencing Testicular Dissociation/Digestion Outcomes

While the vast majority of publications clearly report enzymatic digestion protocols with fixed incubation timings, only one evaluated the progress of digestion by microscopy and the physical presence of remaining testicular tissue and continued until all remaining tissue was digested. This study stopped the enzymatic digestion protocol when the contorted seminiferous tubules became soft and loose and released a “great number” of cells into the solution without specification about exact cell numbers [47]. This strategy resulted in 4.86 ± 1.19 × 10^6^ cell yield after digestion with 91.07 ± 2.16% viability. Unfortunately, start material weight was not reported, thus hampering comparison with protocols using fixed incubation times [47].

**Table 3:** Mechanical dissociation/enzymatic digestion protocols applied on human testicular tissue.

**Mechanical Dissociation Applied to Human Testicular Tissue**					
**Reference**	**Age of Donor**	**Medical Diagnosis/Tissue Source/Anatomopathology** **Number of Donors (*n*)** **Tissue Weight/Size**	**Fresh/Frozen**	**Dissociation Protocol**	**Main Outcomes**
Kurpisz et al., 1988 [35]	32–38 years	Azoospermia *n* = NA	NA	Battery of chilled razor blades (automated)	Single-cell suspension containing spermatocytes, spermatids and 20–25% extensively damaged Sertoli cells
Schneider et al., 2015 [33]	NA	Sex reassignment surgery *n* = 76 (500 mg)	Fresh *n* = 6 Frozen *n* = 7	Medimachine dissociation (automated) for 15 min	Mechanical dissociation of cryopreserved tissue resulted in significantly higher total cell yield compared to enzymatic digestion 38.04% vs. 15.72% viability after mechanical dissociation using fresh or cryopreserved tissue, respectively 75.93% vs. 73.99% viability after enzymatic digestion using fresh or cryopreserved tissue, respectively 2-fold lower spermatogonia (UTF1), 1.5-fold lower germ cell (MAGEA4), 2-fold lower Sertoli cell (VIM), and 5-fold lower Peritubular Myoid cell (ACTA2) expression when Medimachine dissociation was applied on fresh tissue compared to enzymatic digestion 7.95 ± 4.38% and 26.92 ± 6.52% SALL4+ cells when cryopreserved tissue was enzymatically digested compared to mechanical dissociation 19.58 ± 7.88% and 27.33 ± 9.49% SALL4+ cells when fresh tissue was enzymatically digested compared to mechanical dissociation
**Enzymatic Digestion Applied to Human Testicular Tissue**					
**Reference**	**Age of Donor**	**Medical Diagnosis/Tissue Source/Anatomopathology** **Number of Donors (*n*)** **Tissue Weight/Size**	**Fresh/Frozen**	**Digestion Protocol**	**Main Outcomes**
Berensztein et al., 1992 [48]	Prepubertal	Cadaver testis *n* = 7	Fresh	Step 1: 5 KU/mL DNase + 1.182 × 10^3^ U/mL collagenase at 37 °C for 20 min Sedimentation for 5 min Centrifugation Step 2: 15 KU/mL DNase + 1.970 × 10^3^ U/mL collagenase at 37 °C for 20 min Filter 0.4 mm	1.13 ± 0.40 × 10^8^ cell yield/g after 1st step of digestion 2.45 ± 0.32 × 10^8^ cell yield/g at the end of digestion
Brook et al., 2001 [49]	22–35 years	Biopsy with normal spermatogenesis *n* = 11 Orchiectomy whole testes *n* = 8	Fresh	Mincing (3 × 3 × 3 mm^3^) Step 1: 1 mg/mL collagenase I at 37 °C for 12–20 min Filter 120 µM Step 2: 6 µg/mL bovine pancreatic trypsin + 2 mM EDTA + 16 µg/mL ovine hyaluronidase + 0.4 µg/mL DNase I + 0.2 mM sodium pyruvate at 37 °C for 12–15 min 500 µg/mL Soybean trypsin inhibitor Centrifugation 500× *g* at 4 °C for 10 min	66% viability after digestion
Liu et al., 2011 [47]	Fetal 6–7 months	Miscarriage *n* = Not available (NA)	Fresh	Mincing Step 1: 1 mg/mL collagenase I at 37 °C for 10 min Centrifugation Supernatant removed Step 2: 0.25% Trypsin at 37 °C for 10–15 min Centrifugation Filter 200 mesh	4.86 ± 1.19 × 10^6^ cell yield after digestion 91.07 ± 2.16% viability after digestion
Nowroozi et al., 2011 [50]	25–52 years	Non-obstructive azoospermia (NOA) *n* = 47 (100–200 mg/fragment)	Fresh	0.5 mg/L collagenase + 0.5 mg/L Trypsin + 0.5 mg/L hyaluronidase + 0.05 mg/L DNase for 20 min at 37 °C with agitation	93.40 ± 5.04% viability after digestion
Izadyar et al., 2011 [51]	NA	Obstructive azoospermia (OA) testicular sperm extraction (TESE) *n* = 29 Orchiectomy with normal spermatogenesis *n* = 2	Fresh	Step 1: 1 mg/mL collagenase A + 10 U/mL DNase at 37 °C for 15 min Gravity sedimentation and discard of supernatant Step 2: 1.5 mg/mL collagenase A + 1.5 mg/mL hyaluronidase V + 0.5 mg/mL Trypsin + 10 U/mL DNase at 37 °C for 20 min Filter Centrifuge 400× *g* for 10 min	0.5 × 10^6^ cell yield after digestion 87% viability after digestion
Mirzapour et al., 2011 [52]	28–50 years	Azoospermia (maturation arrest) *n* = 20 (100–200 mg/patient)	Fresh	Step 1: 0.5 mg/mL Trypsin + 0.5 mg/mL Hyaluronidase + 0.05 mg/mL DNase at 37 °C for 20 min Centrifugation 112 Relative centrifugal force (RCF) for 4 min Wash Dulbecco’s modified eagle medium (DMEM) Step 2: 0.5 mg/mL Trypsin + 0.5 mg/mL Hyaluronidase + 0.05 mg/mL DNase at 37 °C for 5 min Centrifugation 542 RCF for 4 min at 37 °C Filter 70 µM nylon	93.40 ± 5.04% viability after digestion
Koruji et al., 2012 [53]	32–50 years	NOA TESE *n* = 20	Fresh	Mincing Step 1: 1 mg/mL collagenase I + 1 mg/mL Hyaluronidase + 1 mg/mL Trypsin + 0.05 mg/mL DNase at 37 °C for 30 min with shaking and pipetting Centrifugation 2 min 1100 revolutions per minute (rpm) 3 × wash in DMEM Step 2: repetition step 1 for 30–45 min Filter 40 µM	≥92% viability after 24 h differential plating
Riboldi et al., 2012 [54]	NA	OA *n* = 9 NOA TESE *n* = 11	Fresh	Mincing (1 mm^3^) Step 1: 1000 IU/mL collagenase IA for 20 min at 37 °C on shaker Step 2: TrypLE select for 10 min at 37 °C on shaker Filter 50 µM Centrifugation 1000 rpm for 5 min	75% viability after digestion OA: 4.16 ± 4.90 × 10^6^ cell yield after digestion NOA: 1.99 ± 1.97 × 10^6^ cell yield after digestion
Zohni et al., 2012 [55]	39–50 years	OA *n* = 18 (mean biopsy weight 67.1 ± 8.3 mg/patient)	Fresh	Step 1: 1 mg/mL collagenase I + 1 mg/mL collagenase IV + 1 mg/mL Hyaluronidase + 1 mg/mL DNase I at 33 °C for 15 min with periodic shaking Centrifugation 500× *g* for 5 min Step 2: 0.5 mg/mL Trypsin + 1 mg/mL DNase I at 33 °C for 5 min	8.6 ± 0.4 × 10^4^ cell yield/mg after digestion 5.8 × 10^6^ cell yield/patient 95.5 ± 1.7% viability after digestion
Pacchiarotti et al., 2013 [46]	25–40 years	Sex reassignment surgery *n* = 5	Fresh Frozen	Mincing Liberase (0.3 U/mL collagenase I and II + 1000 U/mL Thermolysin) at 37 °C shaking 110 RPM for 1.75 h Filter 100 µm centrifugation 400 × *g* for 5 min at 4 °C	Fresh: 42.5 ± 9.3 × 10^6^ cell yield/g with 90.1 ± 1.3% viability after digestion Fresh: 0.6 ± 0.1 × 10^6^ spermatogonia stem cell (SSEA-4+), 1.6 ± 0.5 × 10^6^ Leydig cell (LHR+) and 16.6 ± 4.1 × 10^6^ germ cell (VASA+) cell yield/g after digestion Frozen 15.9 ± 4.4 × 10^6^ cell yield/g with 74.0 ± 2.2% viability after digestion Frozen: 0.3 ± 0.1 × 10^6^ Spermatogonial stem cell (SSEA-4+), 22 ± 0.9 × 10^6^ Leydig cell (LHR+) and 10.9 ± 3.3 × 10^6^ germ cell (VASA+) cell yield/g after digestion
Kossack et al., 2013 [56]	NA	Biopsy with normal spermatogenesis *n* = 4 Klinefelter patients *n* = 3	Fresh	Step 1: 1 mg/mL collagenase IA at 37 °C for 30 min Centrifugation 438 × *g* for 5 min Remove supernatant Step 2: 4 mg/mL Trypsin + 2.2 mg/mL DNase I for 10 min at 37 °C	2.91 ± 1.21 × 10^6^ cell yield after digestion of normal spermatogenesis samples 2.87 ± 2.03 × 10^6^ cell yield after digestion of Klinefelter patient samples
Zheng et al., 2014 [45]	13–40 years	Cadaver testis *n* = NA (0.5–2 g/experiment)	Fresh Frozen	Mincing Step 1: 1 mg/mL collagenase IV + 0.7 mg/mL DNase in HBSS at 37 °C for 15 min Step 2: 0.25% Trypsin/EDTA + 0.7 mg/mL DNase in HBSS at 37 °C with periodic rocking for 5 min Filter 40 µM	Fresh: 29 ± 16 × 10^6^ cell yield/g after digestion Frozen: significant lower yield after digestion, exact cellular yield not reported
Guo et al., 2015 [57]	22–35 years	OA *n* = 50	Fresh	Step 1: 2 mg/mL collagenase IV + 1 µg/mL DNase I at 34 °C for 15 min Step 2: 4 mg/mL collagenase IV + 2.5 mg/mL hyaluronidase + 2 mg/mL trypsin + 1 µg/mL DNase I	≥98% viability after overnight differential plating
Jabari et al., 2023 [58]	15, 21, and 26 years old	Cadaver testis *n* = 3	Fresh	Mincing Step 1: 1 mg/mL collagenase I + 1 mg/mL hyaluronidase + 1 mg/mL Trypsin + 0.05 mg/mL DNase at 37 °C with 150 cycles/min shaker for 30 min Centrifugation 1100 rpm for 4 min Wash in DMEM Step 2: Repetition step 1 for 25 min Filter 40 µm	>91% viability after digestion
Nikmahzar et al., 2023 [59]	28, 32, and 44 years	Cadaver testis *n* = 3	Fresh	Step 1: 1 mg/mL collagenase IV + 1 mg/mL hyaluronidase at 37 °C for 10 min at 150 cycles/min shaken Centrifugation at 1100 rpm for 10 min Step 2: 1 mg/mL collagenase + 0.5 mg/mL DNase I + 1 mg/mL hyaluronidase at 37 °C for 10 min Filter 100 µM and 40 µM	70% viability after digestion

ACTA2, actin alpha 2; DMEM, Dulbecco’s modified eagle medium; EDTA, ethylenediaminetetraacetic acid; HBSS, Hanks’ balanced salt solution; IU, international unit; KU, Kunitz unit; LHR, luteinizing hormone receptor; MAGEA4, melanoma-associated antigen 4; NA, not available; NOA, non-obstructive azoospermia; OA, obstructive azoospermia; RCF, relative centrifugal force; RPM, revolutions per minute; SALL4, Sal-like protein 4; SSEA-4, stage-specific embryonic antigen-4; TESE, testicular sperm extraction; UTF1, undifferentiated embryonic cell transcription factor 1; VIM, vimentin.

### 3.2. Testicular Cell Selection/Enrichment, and Characterization

Selection or enrichment of testicular cells is essential for research purposes, as the human testis contains a highly heterogenous population of SCs, GCs, Leydig cells (LCs), and Peritubular Myoid cells (PTMCs) each at different developmental stages. As some cell types are scarce, and can be masked by more abundant cell subpopulations, obtaining the purest fraction of a certain cell of interest is pivotal for downstream applications. In particular, the cells of focus for fertility restoration approaches, the undifferentiated GCs, are present in very low proportions ranging from 1% undifferentiated GCs in human ITT from newborns of 2–7 days old [60] to 3–4% in the biopsy of a 1-year- and 7-year-old boy [61].

Table 4 summarizes selection/enrichment strategies and their outcomes for all major testicular cell types.

#### 3.2.1. Sertoli Cells

Many studies have tried to isolate the SC, a known key player in both the architecture and the function of the testes. Among these, SCs are known to regulate the formation of the blood–testis barrier through functional and dynamic tight junctions creating an exclusive immunogenic environment to protect GCs from own immune cell attacks [62]. Apart from aiming to isolate SCs to study their morphology or transcriptome, this is also performed to use them as feeder cells, as spermatogonia require feeder-cell-based substrates to promote their survival and proliferation in vitro [47,52,63]. Methods identified in the literature to isolate SCs are based on density gradients, differential plating, flow cytometry or a combination of techniques to further purify the obtained SC fraction.

##### Selection/Enrichment of SCs by Density Gradient Separation

Silica-based gradient separation preparations such as Percoll are commonly used in cell selection applications. Percoll uses silica particles coated with polyvinylpyrrolidone to create a low viscosity density gradient medium in which cells can be separated after centrifugation [47,64]. SCs, identified morphologically, were isolated from 6-to-7-month-old fetal ITT by recuperating the bands of 19–27% and 35–43% Percoll. Unfortunately, the efficiency of the technique remains unknown as neither cellular yield nor viability were reported [47].

##### Selection/Enrichment of SCs by Differential Plating

SCs are well characterized by their ability to adhere to (un)-coated plastics, a characteristic that GCs possess with much less affinity [65]. Therefore, most studies aiming to isolate or enrich high-purity SC fractions perform differential plating strategies by separating adherent and floating cell fractions. An attachment rate of 63.33 ± 8.76% [65] and a mean number of adherent cells of 1.93 ± 1.16 × 10^5^ somatic cells were obtained with adult tissue [66]. Enzymatic digestion of adult obstructive azoospermia (OA) testicular biopsies followed by 24 h incubation onto plastic in standard medium-separated floating GCs from adherent SCs. This approach allowed the enrichment of SCs by 95% purity and 98% viability [57]. Another study incubating the single-cell suspension for 48 h in standard medium characterized the SCs morphologically and concluded 90% SC purity with over 95% viability [67]. Mirzapour et al., 2011 [52], used coated plastics to even further facilitate the adherence of SCs by using Datura Stramonium Agglutinin (DSA)-coated plastics and while purity reached 95%, it did not differ significantly from uncoated plastic differential plating.

##### Selection/Enrichment of SCs by Combinatory Strategies

Adult human SCs could be successfully isolated after multiple enzymatic digestion steps, centrifugations, multiple gravity sedimentation steps with supernatant discarded in each step and following long-term culture. The fractions were analyzed based on morphology [68,69] or based on SC-specific marker identification resulting in a pure SC fraction of 95% SCs [70] with viability ranging from 75 to 95%.

#### 3.2.2. Leydig Cells

LCs are found adjacent to the seminiferous tubules and act to stimulate spermatogenesis by producing androgens [71]. Only one study examined the theoretical yield of LCs after enzymatic digestion. The average theoretical number of LCs per adult testis was calculated to be 139.12 ± 81.89 × 10^6^, with minimum and maximum values of 43.20 × 10^6^ and 351.28 × 10^6^, respectively [72], which account for 2–4% of the total testicular cell population [73].

##### Selection/Enrichment of LCs by Density Gradient Separation

Discontinuous Percoll gradients are commonly used to isolate and enrich LCs from enzymatically digested single-cell suspensions [72,74,75,76]. Only one study investigated the purity of LCs in all 3 layers formed between 25 and 70% and showed a 3β-HSD+ LC enrichment of 12–28%, 48–70%, and 30–56% in band 1 (25–45% Percoll), 2 (45–60% Percoll), and 3 (60–70% Percoll), respectively [74]. Despite having the highest enrichment of LCs in band 2, it only recovered 4–18% of all LCs, as 77–95% of all LCs are found in band 1 [74]. Another study collected the 34% and 40% bands, yielding a 95% enriched 3β-HSD+ viable LC suspension. Although the exact purity was not reported, they claimed to have obtained 500.000 LCs/mL [75]. When collecting all cells between 34 and 60%, a highly viable (±94%) LC-enriched fraction was obtained ranging from 80 to 83% 3β-HSD+ LCs. Of note, they identified a difference in staining intensity among the same LC-enriched suspensions indicating a heterogeneity among the LC populations [76]. Another study recovered LCs in the interphase of 35–60% Percoll and yielded 60–77% pure 3β-HSD+ LCs [72]. Furthermore, one study identified a recovery of only 6–12% of the total LC population by collecting all bands formed between 25 and 70% Percoll [74].

##### Selection/Enrichment of LCs by Flow Cytometry

To obtain LCs by sorting, some promising membrane markers have been identified. In their study, Xia et al., 2020 [77] successfully isolated stem Leydig cells (sLCs) by fluorescent-activated cell sorting (FACS), using the presence of endosialin receptors. The endosialin+ sorted cells account for 0.31 ± 0.03% of the total cell population, though purity was not reported. After one week of culture, 99.30 ± 0.32% of the FACS-sorted cells were still endosialin+. Characterization of the obtained endosialin+ fraction after one week of culture revealed a purity of ≥98% using well-established LC markers such as PDGFR-α, NGFR, and Nestin. Furthermore, these endosialin+ cells were xenotransplanted for 4 weeks and were exclusively found in the interstitial area keeping their PDGFR-α expression, while a small proportion of the grafted cells expressed adult LC markers (3β-HSD, CYP11A1, CYP17A1, and StAR), indicating their differentiation potential into LCs.

#### 3.2.3. Peritubular Myoid Cells

Testicular PTMCs are smooth, muscle-like cells that surround the seminiferous tubules forming the blood–testis barrier together with SCs and will support sperm movement through contractile activity [78]. Only a few studies attempting to isolate and obtain highly pure PTMC fractions are reported. Thanks to their migration capacities, a unique selection method, not reported for other testicular cell types, the explant growth culture or tubule crawling method, has been applied to isolate PTMCs from testicular tissue [79,80,81,82].

##### Selection/Enrichment of PTMCs by Explant Growth Culture

In this approach, testicular fragments are cultured for 1 to 3 weeks in defined medium containing recalcified human serum to promote cell migration allowing PTMCs to grow out of the biopsies and attach to the culture plate [81,82,83,84]. Results suggested that the PTMC fraction host putative sLCs as 79–90% of all isolated cells expressed sLC marker PDGFRα and pluripotency marker Nanog of which almost all had positive PTMC marker α-SMA and LC marker StAR staining; this finding was further confirmed by reverse transcription polymerase chain reaction (RT-PCR) in four independent human adult donors using sLC and PTMC markers [82]. This indicates that explant growth culture inevitably leads to contamination with other somatic cells and thus is unable to obtain highly pure PTMCs [79].

##### Selection/Enrichment of PTMCs by Flow Cytometry

To overcome somatic cell contamination, Han et al., 2025 [79], identified ITGA9 as being only expressed on PTMCs while NGFR is a commonly expressed membrane receptor for both adult LCs and PTMCs, opening the way for flow cytometry. Indeed, by sorting the ITGA9+/NGFR+ fraction, this study successfully isolated ≥95% pure PTMC fractions being positive for well-characterized PTMC markers in combination with the absence of LC and SC markers.

#### 3.2.4. Germ Cells

Different approaches such as magnetic-activated cell sorting (MACS), differential plating, flow cytometry, and Percoll gradient have been applied to isolate and select SSCs and undifferentiated GCs. To date, no SSC-specific marker has been identified for any species, but the combination of multiple markers is able to identify spermatogonial cell types in the human testis of different maturation statuses. Thanks to improvement in sequencing assays, nowadays, analysis at the single-cell level revealed some promising new markers for putative SSCs [85,86,87].

##### Selection/Enrichment of SSCs by MACS

To date, SSCs can only be identified using the xenotransplantation assay with downstream verification of colonization capacities of SSCs. A proof of principle can be found in the study of Nickkholgh et al., 2014 [88], in which they tested SSC enrichment after a 50 day culture and MACS by analyzing colony formation efficiency after xenotransplantation. They showed that ITGA6+ sorted cells had the highest number of colony formations compared to Human Leukocyte Antigen class I (HLA)−/GPR125+, GPR125+, and HLA−/ITGA6+ with 7.1; 3.9; 3.9 and 2-fold SSCs enrichment, respectively. Using cell number (not number of colonies formed) as an outcome, other studies have shown that MACS for the cell-surface protein SSEA-4, resulting in 90.2% SSEA-4+ cells, enriches human adult testicular cells for SSC activity by ≥40-fold [51].

##### Selection/Enrichment of SSCs by Flow Cytometry

Recently, a comparative RNA-sequencing revealed a 38-fold SSC activity in PLPPR3+ FACS-sorted fractions compared to unsorted testicular fractions. However, PLPPR3 was identified to be a transmembrane protein with half of the PLPPR3+ cells having PLPPR3 present on the membrane which limits the use of PLPPR3 to sort SSCs [89].

##### Selection/Enrichment of GCs by Differential Plating

Separation of GCs by their weak ability to attach to uncoated plastic is a common approach to obtain GC-enriched fractions [52,58,65,90,91]. In their study, Medrano et al., 2016 [65], investigated the number of adult spermatogonia per cm^2^ in unsorted and 24 h-incubated differential plated fractions. They found an enrichment of GCs in the unattached differential plated fraction compared to the attached differential plated fraction with 50 and 8 VASA+/UTF1+ undifferentiated GCs/cm^2^, respectively. Other approaches used protein-coated plastic dishes, such as the commonly used DSA-coating, to facilitate the adherence of somatic testicular cells leading theoretically to higher enriched floating GC fractions. As incubating single-cell suspensions for 2–3 h led to a 95% pure GC fraction by recovering the floating cell fraction, this indicates that protein-coated dishes did not result in better outcomes [52].

##### Selection/Enrichment of GCs by Flow Cytometry

To achieve undifferentiated GC selection/enrichment, several cell-specific markers have been used. Among those, epithelial cell adhesion molecule (EPCAM) and HLA resulted in the promising enrichment of putative SSCs. In the study of Medrano et al., 2016 [65], cryopreserved adult testicular cells were enzymatically digested and a FACS-sorted GC enriched fraction was compared with an unsorted single-cell suspension. The comparison revealed that GC enrichment by HLA negative/EPCAM positive resulted in an enrichment of VASA+/UTF1+ human putative SSCs by 14%; unfortunately, no xenotransplantation assay was performed to further characterize the obtained cells.

##### Selection/Enrichment of GCs by MACS

Alternatively to FACS, sorting cells by magnetic bead pre-labeling of the cell of interest proved to be effective. He et al., 2010 [92], isolated GPR125+ putative SSCs from adult donors by MACS and obtained 95% pure GPR125+ fractions which showed after 2-week culture a 5-fold increase in cell numbers retaining ITGA6+, GFRA1+, and THY1+ expression and phenotypical characteristics of putative human SSCs.

##### Selection/Enrichment of GCs by Combinatory Approaches

As all these flow cytometric sorting strategies did not lead to optimal outcomes, further effort was put into obtaining GC-sorted fractions. Von Kopylow et al., 2016 [93], sorted patient-derived single-cell suspensions from adult tissue containing normal spermatogenesis and maturation arrest by MACS followed by micromanipulation cell-picking of only the FGFR3+ fraction. A 100% pure putative SSC population could be obtained with 100% of the picked cells expressing UTF1 and 95% viability, though stem cell characteristics have not been tested and not all UTF1+ cells were recovered as the negative sorted fraction still contained 1% UTF1+ cells. Furthermore, human fetal undifferentiated GCs could be isolated using Percoll density gradient separation by recovering all cells in layers formed between 27 and 35% in combination with 24 h culture and followed by flow cytometry. This combinatory approach resulted in 86.7% purity confirmed by the presence of transcription factor membrane marker OCT4 [47]. Alternatively, STA-PUT enrichment based on sedimentation and microscopical evaluation was found to lead to promising viability and purity of enriched testicular cell suspensions. Testes from OA patients were enzymatically digested and GCs were first enriched by differential plating. This enriched single-cell suspension was further loaded on the STA-PUT gravity apparatus to separate and enrich all GC types. In brief, the GC-enriched single-cell suspension was mixed with multiple bovine serum albumin concentrations to form a gradient. At the end, 45 fractions each containing cells of similar size were formed and fractions containing cells with similar morphology were pooled manually to separate different GC types. In general, spermatogonia could be enriched with a purity of 90% based on immunohistochemistry and RT-PCR using well-characterized specific undifferentiated GC markers and led to a viability ≥98%. Spermatogonia, microscopically identified as cells with spherical, large round or ovoid nuclei, diameter 9–12µM and high ratio of nucleus to cytoplasm, were found in fraction 10–15 [94].

**Table 4:** Selection/enrichment strategies applied on human testicular tissue.

**Sertoli Cell Selection/Enrichment Strategies Applied on Human Testicular Tissue**					
**Reference**	**Age of Donor**	**Medical Diagnosis/Tissue Source/Anatomopathology** **Number of Donors (n)** **Tissue Weight/Size**	**Fresh/Frozen**	**Selection/Enrichment Strategy After Enzymatic Digestion** **+ Verification Technique**	**Main Outcomes**
Lipshultz et al., 1982 [69]	Adult	Sex reassignment surgery *n* = NA	Fresh	Differential plating and culture for 45 days Morphological identification	>95% pure Sertoli cell culture after 45 days 75–85% cell viability after 45 days
Teng et al., 2005 [67]	28–42 years	Cadaver testis *n* = NA	Fresh	Differential plating and culture for 28 days Morphological identification	>90% pure Sertoli cell culture after 28 days >95% cell viability after 28 days
Chui et al., 2011 [68]	12–36 years	Cadaver testis *n* = 7	Fresh	Differential plating and culture for 20 days Morphological identification + Flow cytometry (GATA-4/SOX9) + RT-PCR (*SCF*, *GDNF* and *BMP4*)	≥95% pure Sertoli cells expressing Sertoli cell (*SCF*, *GDNF*, and *BMP4*) markers with 90% viability after 20 days culture
Mirzapour et al., 2011 [52]	28–50 years	Azoospermia (maturation arrest) *n* = 20 (100–200 mg/sample)	Fresh	Differential plating to (un)-DSA coated dishes and culture for 72 h ICC (VIM)	>95% pure Sertoli cells after 72 h culture No statistically significant difference in Sertoli cell purity when using DSA-coated dishes compared to uncoated dishes
Riboldi et al., 2012 [54]	NA	Obstructive azoospermia (OA) *n* = 9 Non-obstructive azoospermia (NOA) TESE *n* = 11	Fresh	Differential plating and culture	95% pure Sertoli cell after differential plating
Guo et al., 2015 [57]	22–35 years	OA *n* = 50	Fresh	Differential plating and overnight culture RT-PCR (*WT-1*, *GATA-4*, *GATA-1*, *GDNF*, *BMP4*, *SCF*, *FGF2*, *EGF*, *FSHR*, *AR* and *ABP*) + ICC (WT-1, GDNF, SCF, BMP4, VIM, PCNA, and GATA-4)	98% Sertoli cell viability after overnight culture 95% pure Sertoli cell culture <5% of the enriched cells expressed Peritubular Myoid cells (α-SMA) or Leydig cell (CYP11A1) markers
Gaur et al., 2018 [70]	26–56 years	Cadaver testis *n* = 5	Fresh	Multiple gravity sedimentation steps ICC (GATA-4)	>95% pure Sertoli cell culture
**Leydig Cell Selection/Enrichment Strategies Applied on Human Testicular Tissue**					
**Reference**	**Age of Donor**	**Medical Diagnosis/Tissue Source/Anatomopathology** **Number of Donors (*n*)** **Tissue Weight/Size**	**Fresh/Frozen**	**Selection/Enrichment Strategy after Enzymatic Digestion** **+ Verification Technique**	**Main Outcomes**
Simpson et al., 1987 [74]	57–85 years	Orchiectomy for prostatic carcinoma *n* = 10 (Testis fragment weight used 5–8 g/patient)	NA	Discontinuous Percoll gradient Immunohistochemistry (3β-HSD)	After DPG 3 bands were obtained Band 1: 95–97% of all testicular cells, 12–28% pure Leydig cells accounting for 77–95% of all Leydig cells, 10.6–16.6 × 10^6^ Leydig cell yield Band 2: 2–4% of all testicular cells, 48–70% pure Leydig cells accounting for 4–18% of all Leydig cells, 0.7–5.7 × 10^6^ Leydig cell yield Band 3: 0.7–1.5% of all testicular cells, 30–56% pure Leydig cell yield accounting for 1–6% of all Leydig cells, 0.3–1.9 × 10^6^ Leydig cell yield Overall, 11.6–24.2 × 10^6^ Leydig cell yield
Qureshi et al., 1993 [72]	54–89 years	Orchiectomy for prostatic carcinoma *n* = 27 (Testes paired weight 6.6–59.48 g)	Fresh	Discontinuous Percoll Gradient Immunohistochemistry (3β-HSD)	60–77% pure Leydig cells obtained after DPG 139.12 ± 81.89 × 10^6^ Leydig cell yield/testis
Sivakumar et al., 2006 [75]	60–70 years	Orchiectomy for prostatic carcinoma *n* = NA	Fresh	Discontinuous Percoll Gradient Immunohistochemistry (3β-HSD)	95% cell viability after DPG 0.5 × 10^6^ Leydig cell yield/mL after DPG
Bilinska et al., 2009 [76]	60–67 years	Orchiectomy for prostatic carcinoma *n* = 4	Fresh	Discontinuous Percoll Gradient Morphological identification ICC (3β-HSD/LHR)	DPG 34–60%: 80–83% pure Leydig cells 94% Leydig cell viability
Zhang et al., 2017 [73]	18 and 19 years 23, 25, 28, 32 years	Cadaver testis n = 2 OA *n* = 4	NA	FACS (p75)	p75 sorted stem Leydig cells account for 1.79% of the total cell population.
Xia et al., 2020 [77]	56–60 years 57–67 years	Cadaver testis *n* = 2 Orchiectomy for prostatic carcinoma *n* = 2	NA	FACS (Endosialin) ICC (PDGFRα, NGFR, and Nestin)	Endosialin+ sorted stem Leydig cells accounted for 0.31 ± 0.03% of the entire cell population with >98% expressing Leydig cell (PDGFRα, NGFR, and Nestin) markers
Han et al., 2025 [79]	NA	OA TESE *n* = 3	Fresh	FACS (ITGA9/NGFR) ICC (3β-HSD, α-SMA, StAR)	ITGA9+/NGFR−sorted cells account for 0.2% of the total cell population, ITGA9+/NGFR+ sorted cells account for 0.68% of the total cell population and 95% expressed Peritubular Myoid cell (α-SMA) marker ITGA9−/NGFR + sorted cells were Leydig cell (3β-HSD, StAR) marker positive
**Peritubular Myoid Cell Selection/Enrichment Strategies Applied on Human Testicular Tissue**					
**Reference**	**Age of Donor**	**Medical Diagnosis/Tissue Source/Anatomopathology** **Number of Donors (*n*)** **Tissue Weight/Size**	**Fresh/Frozen**	**Selection/Enrichment Strategy** **+ Verification Technique**	**Main Outcomes**
Albrecht et al., 2006 [81]	29, 32, 32, 34, 35, 36, 40, 41, 46, 47 years	OA with normal spermatogenesis *n* = 8 Varicocele with slightly reduced spermatogenesis *n* = 2	Fresh	Explant growth culture Morphological identification + immunohistochemistry (FSH, LH-r, α-SMA, THY1) + RT-PCR (*Tryptase*, *Histamine*)	Peritubular Myoid cells become visible after 1–2 weeks and expressed Peritubular Myoid cell (α-SMA, *Tryptase* and *Histamine*) and germ cell (THY1) markers while not expressing Sertoli cell (FSH) and Leydig cell (LH-r) markers
Landreh et al., 2014 [82]	31–52 years	OA NOA *n* = NA	NA	Explant growth culture Immunohistochemistry (PDGFR-α, α-SMA, StAR)	Almost all outgrowth cells expressed Peritubular Myoid marker (α-SMA) and Leydig cell (StAR) marker and 79–90% of them were Leydig cell (PDGFR-α) marker positive
Rolland et al., 2019 [84]	Average 51 years Average 80 years	Cadaver testis *n* = 8 Orchiectomy *n* = 9	NA	Explant growth culture	Peritubular Myoid cells grow out after 2–3 weeks
Han et al., 2025 [79]	NA	OA TESE normal spermatogenesis *n* = 3	Fresh	FACS (ITGA9/NGFR) ICC (3β-HSD, α-SMA, StAR)	ITGA9+/NGFR−sorted cells account for 0.2% of the total cell population, ITGA9+/NGFR+ sorted cells account for 0.68% of the total cell population and 95% expressed Peritubular Myoid cell (α-SMA) marker ITGA9−/NGFR+ sorted cells were Leydig cell (3β-HSD, StAR) marker positive
**Germ Cell Selection/Enrichment Strategies Applied on Human Testicular Tissue**					
**Reference**	**Age of Donor**	**Medical Diagnosis/Tissue Source/Anatomopathology** **Number of Donors (*n*)** **Tissue Weight/Size**	**Fresh/Frozen**	**Selection/Enrichment Strategy after Enzymatic Digestion** **+ Verification Technique**	**Main Outcomes**
He et al., 2010 [92]	16–58 years	Cadaver testis *n* = 5	Fresh	Differential plating + MACS (GPR125) ICC (GPR125)	6 × 10^6^ germ cell yield/g after differential plating 3 × 10^4^ germ cell yield after MACS >95% pure germ cell after MACS cells could be proliferated 5-fold during 14-day culture while retaining phenotypical characteristics of SSCs
Liu et al., 2011 [47]	Fetal 6–7 months	Miscarriage *n* = NA	Fresh	Discontinuous Percoll gradient followed by 3 h-differential plating ICC (SSEA-4, OCT4)	86.7% pure OCT4+ germ cell after DPG and differential plating of which the majority expressed germ cell marker SSEA-4
Izadyar et al., 2011 [51]	NA	OA TESE *n* = 29 Orchiectomy with normal spermatogenesis *n* = 2	Fresh	MACS (SSEA-4) RT-PCR (*c-KIT*, *GFRα1*, *PLZF*, *c-RET*, *GPR125*, *Dppa5*, and *hTERT*) + Xenotransplantation	SSEA-4+ sorted germ cells account for 13.3 ± 1.4% of the entire single-cell suspension and had significantly higher expression of SSC-specific genes compared to SSEA-4- sorted cells SSEA-4+ sorted cells showed 40–50-fold HNP+ cells after xenotransplantation compared to unsorted single-cell suspension xenotransplantation
Mirzapour et al., 2011 [52]	28–50 years	Azoospermia (maturation arrest) *n* = 20 (100–200 mg/patient)	Fresh	Differential plating to DSA-coated dishes for 2–3 h and culture ICC (OCT4) + RT-PCR (*Oct4*, *Nanog*, *Piwil2*, *Stra8*, *Vasa*, *Bax,* and *DMC1*) + Xenotransplantation	95% pure germ cell after differential plating and SSC colonies expressed key germ cell markers after 2 weeks of culture
Nickkholgh et al., 2014 [88]	NA	Orchiectomy (prostate cancer) *n* = 2	Frozen	Culture for 50 days followed by MACS (GPR125 or ITGA6 in combination with HLA) qRT-PCR (*ID4*) + Xenotransplantation	5.3 ± 3.8% ITGA6+, 1.99 ± 1.5% HLA−/ITGA6+, 1.89 ± 0.9% GPR125+, and 2.33 ± 0.7% HLA−/GPR125+ after MACS higher expression of undifferentiated germ cells (*ID4*) in ITGA6+ and HLA−/GPR125+ sorted fraction Xenotransplantation: No significant difference among GPR125+/HLA− or ITGA6+/HLA− or GPR125+ or ITGA6+ MACS sorted fractions regarding SSC colony formation
Smith et al., 2014 [95]	Adult	Normal spermatogenesis *n* = 13	NA	FACS (SSEA-4, THY1) RT-PCR (*ZBTB16*, *GFRa1*), ICC (DAZL, VASA)	SSEA-4+ sorted germ cells expressed undifferentiated germ cell (DAZL, VASA) markers and had significantly higher undifferentiated germ cell (1.9-fold ZBTB16, 10-fold GFRa1, and 3-fold GPR125) marker expression compared to THY1+ sorted germ cell fractions
Von Kopylow et al., 2016 [93]	Adult	OA with normal spermatogenesis *n* = 37 Meiotic arrest *n* = 3 (30 mg/testis)	Fresh	MACS (FGFR3) + Micromanipulation cell-picking Morphological identification + ICC (UTF1)	54–138 and 220–280 UTF1+ undifferentiated germ cell yield after micromanipulation in normal spermatogenesis and meiotic arrest patients, respectively 100% pure UTF1+ undifferentiated germ cells compared to 1–2% UTF1+ cells in unsorted control with viability 95%
Medrano et al., 2016 [65]	Adult	Bilateral orchiectomy (prostate cancer, normal spermatogenesis) *n* = 3	Frozen	Differential plating 24 h or FACS (HLA−/EPCAM+) ICC (VASA/UTF1)	27% pure VASA+/UTF1+ undifferentiated germ cells in sorted compared to 13% unsorted fraction 112 undifferentiated germ cell/cm^2^ yield compared to unsorted cells and differentially plated cells 61 and 49 undifferentiated germ cells/cm^2^, respectively 50 and 8 VASA+/UTF1+ undifferentiated germ cells/cm^2^, in floating and adherent fraction after 24 h differential plating, respectively
Tan et al., 2020 [89]	30–50 years	vasectomy reversal *n* = 29	Frozen	FACS (PLPPR3 or KIT) Xenotransplantation	Xenotransplantation: 38-fold SSC-activity enrichment of PLPPR3+ SSCs compared to unsorted cells
Salem et al., 2023 [91]	22, 25, and 28 years	Cadaver testis *n* = 3	Fresh	Differential plating Morphological identification + ICC (PLZF, GFRA1) + RT-PCR (*GFRA1*, *PLZF*, *SCP3*, and *PRM2*) + xenotransplantation	GFRA1+ and PLZF+ SSC colonies were visible after 2 weeks of culture Xenotransplantation: SSC colonies were positive for undifferentiated germ cell markers (PLZF and GFRA1), and differentiated germ cell markers (*SCP3* and *PRM2*) after 8 weeks

3β-HSD, 3 beta-hydroxysteroid dehydrogenase; ABP, androgen binding protein; AR, androgen receptor; α-SMA, alpha-smooth muscle actin; BAX, BCL2 associated X; BMP4, bone morphogenetic protein 4; CYP11A1, cytochrome P450 family 11 subfamily A member 1; DAZL, deleted in azoospermia-like; DMC1, DNA Meiotic recombinase 1; DPG, discontinuous Percoll gradient; DPPA5, developmental pluripotency associated 5; DSA, Datura stramonium agglutinin; EGF, epidermal growth factor; EPCAM, epithelial cell adhesion molecule; FACS, fluorescent-activated cell sorting; FGF2, fibroblast growth factor 2; FGFR3, fibroblast growth factor receptor 3; FSH, follicle stimulating hormone; GATA-1, GATA binding protein 1; GATA-4, GATA binding protein 4; GDNF, glial cell line-derived neurotrophic factor; GPR125, G-protein coupled receptor 125; HLA, human leukocyte antigen; HNP, human nuclear protein; hTERT, human telomerase reverse transcriptase; ICC, immunocytochemistry; ITGA6, integrin subunit alpha 6; ITGA9, integrin subunit alpha 9; LHR, luteinizing hormone receptor; MACS, magnetic-activated cell sorting; NA, not available; NGFR, nerve growth factor receptor; NOA, non-obstructive azoospermia; OA, obstructive azoospermia; OCT4, octamer-binding transcription factor 4; PCNA, proliferating cell nuclear antigen; PDGFR-α, platelet-derived growth factor receptor alpha; PIWIL2, piwi-like RNA-mediated gene silencing 2; PLPPR3, phospholipid phosphatase related 3; PLZF, promyelocytic leukemia zinc finger; PRM2, protamine 2; RT-PCR, reverse transcription polymerase chain reaction; SCF, stem cell factor; SCP3, synaptonemal complex protein 3; SOX9, SRY-Box 9; SSC, spermatogonial stem cell; SSEA-4, stage-specific embryonic antigen-4; StAR, steroidogenic acute regulatory protein; STRA8, stimulated by retinoic acid gene 8; TESE, testicular sperm extraction; THY1, thymocyte differentiation antigen 1; UTF1, undifferentiated embryonic cell transcription factor 1; VIM, vimentin; WT-1, Wilms’ tumor gene 1; ZBTB16, zinc finger and BTB domain-containing protein 16.

### 3.3. Discussion

Research on human testicular tissue or cells is often hampered by low cellular starting numbers in combination with cell death due to dissociation/digestion-induced damage and suboptimal selection and/or enrichment strategies. The current literature, comparing mechanical dissociation protocols or enzymatic digestion protocols is inconclusive as most crucial start material characteristics are not reported. Review results indicate that testicular tissue cryopreservation influences digestion/dissociation outcomes which may hamper comparisons between studies. We may assume that other start material characteristics such as maturity and disease-related tissue alterations may also potentially influence digestion/dissociation outcomes. This further challenges the interpretation of outcomes of the few studies including a control group due to the different tissue characteristics before digestion, such as accumulated ECM products resulting in fibrotic remodeling [96], increased innervation density and numbers of mast cells and macrophages [97,98,99] and lower numbers of SCs, GCs, and, in particular, SSCs [100] compared to normozoospermia age-matched controls.

Furthermore, for similar tissue characteristics, digestion protocols differ in the types of enzymes, concentrations, incubation times, and conditions, thus further precluding the identification of the best enzymatic digestion protocol. When there are no predetermined fixed incubation times, the decision to stop an enzymatic reaction can also have an influence on the resulting single-cell suspension as incomplete digestion may enrich certain cell types in the final suspension at the cost of others that are more difficult to liberate.

Enzymatic digestion demonstrated its superiority compared to mechanical dissociation, in particular for cryopreserved tissue. This is especially important to consider as fertility restoration is based on cryo-stored ITT available in fertility centers worldwide, and as a better preservation of the integrity of the somatic cell fraction was observed for enzymatic digestion compared to mechanical dissociation [33]. The question of which enzymatic digestion protocol should preferably be used and in which condition is still unaddressed. Besides concerns related to differences in start material content or its prior cryopreservation or not, this gap of knowledge in today’s literature is mainly caused by the fact that only few studies report the start volume and/or weight of the tissue in combination with both cell viability and cellular yield, making it impossible to calculate the efficacy of each protocol. Indeed, an example can be found in the study of Sivakumar et al., 2006, [75] in which they reported a LC yield of 500.00 cells/mL; without knowing the start material weight, no normalization of cellular yield per mg of digested tissue can be calculated, thus hampering comparisons with other protocols.

Once single-cell suspensions are obtained, different strategies have been identified to select or enrich specific testicular cell types for downstream reproductive applications such as a safe single-cell transplantation to achieve recolonization of the human testicular niche. So far, attempts to decontaminate testicular tissue by cancer cell sorting resulted in disappointing results with insufficient decontamination while cell culture seemed to be more effective to eliminate cancer cells (for review see [18]). However, final proof of the efficacy of the culture approach is needed based on the possibility that cancer-specific antigen receptor gene targets used for minimal residual disease (MRD)-PCR could be modified during culture and because leukemic cells added to the culture could behave differently from leukemic cells obtained from contaminated testicular tissue. Alternatively, testicular cell selection or a combination strategy with a negative selection of cancer cells could be a promising strategy. Today, to the best of our knowledge, such a strategy with verification of the presence or not of cancer cells, has not been investigated yet. Therefore, this review tried to summarize options to sort testicular cells and report their outcomes to verify if pure single-cell suspensions, indicating absence of cancer cells, could be obtained.

Techniques such as gravity sedimentation, Percoll gradient, and differential plating allowed the selection of highly pure specific cell types, but studies reporting the obtained purity after selection/enrichment in combination with the total recovery number are rarely found. Such data is pivotal in the case of fertility restoration as not only pure single-cell fractions, but also sufficient cell numbers are required for fertility restoration. This is crucial in the case of SSC selection, as these stem cells maintain spermatogenesis and the colonization rate after transplantation is dependent on the number of transplanted SSCs [101]. Before research gaps are addressed, flow cytometry appears the most promising selection technique that led to the highest enrichment in SSCs in studies on cancer cell decontamination of human testicular cell suspensions. Of note, xenotransplantation of FACS-sorted human GC fractions did not result in tumor formation, but the invasive capacity of the cancer cell line that was used is questionable, as in controls where pure cancer cells were xenotransplanted, only a 43% tumor development rate was observed [29]. This highlights the need for further validation of this decontamination strategy. These results indicate that, currently, no testicular tissue digestion and cell selection is able to guarantee the safe autotransplantation of human SSCs when cancer cells are present in testicular tissue. However, this technique was successful in mice based on the absence of tumor development after allotransplantation of FACS-sorted fractions and histological assessment of testes recovered 8 weeks post-allotransplantation [102]. Therefore, further progress may be expected for human samples with combinations of cell markers to better target the cells of interest as single cell sequencing already identified heterogeneity among spermatogonial cell populations [85,86,87,103].

Since this scoping review focused on reproductive purposes starting from ITT, articles only reporting outcomes of dissociation/digestion and/or cell selection/enrichment for differentiated GCs were not included. These papers could contain interesting information; however, the usage of these papers is out of scope for the purpose of this review.

## 4. Conclusions

At present, testicular tissue dissociation/digestion is performed using a plethora of protocols, with no superiority of a particular protocol. The literature indicated that some testicular cell types, in particular the somatic SCs, are more sensitive to dissociation-induced damage. Furthermore, start material characteristics are important, and protocols might need to be adapted according to them. Currently, comparing protocols and their outcomes is hampered by the missing systematic reporting of start material characteristics and corresponding outcomes both at overall and cell-type specific levels. Summarizing tables in this review might help researchers to identify the highest achievements with digestion protocols and downstream selection/enrichment strategies applied to human testicular tissue, and to further cover research gaps towards the clinical application of fertility restoration techniques in cancer patients.

## Figures and Tables

**Table 1:** Testicular cell selection techniques with main outcomes and unaddressed research gaps before clinical application. FACS: Fluorescent-activated cell sorting; GC: Germ cell; LC: Leydig cell; MACS: Magnetic-activated cell sorting; PTMC: Peritubular Myoid cell; SC: Sertoli cell; SSC: spermatogonial stem cell.

Testicular Cell Selection Strategy	Main Achievements	Research Gaps for Clinical Applications
Density gradient separation	95% Leydig cell (LC) purity, 95% viability	Not tested for cancer decontamination purposes Toxicity of silica-based gradients Low cell recovery Limited standardization
Differential plating	>95% Sertoli cell (SC) purity, 98% viability 95% germ cell (GC) purity	Not tested for cancer decontamination purposes Limited standardization Need for identification of optimal protein-coating of culture plates Impossible to isolate specific GC subtypes Limited capacity to achieve cell-type specific separation
Flow cytometry	98% LC purity 95% Peritubular Myoid cell purity 14% GC enrichment 38-fold Spermatogonial stem cell (SSC)-activity No tumor formation after Fluorescent-activated cell sorting (FACS)-sorted xenotransplantation of putative SSCs [29]	Questionable invasive capacities of the cancer cell line used in the decontamination protocol Lack of optimal markers for 100% pure testicular cell selection Additional fluorescent dye removal steps required
Explant growth culture	Peritubular Myoid cells (PTMCs) outgrowth after 2–3 weeks	Not tested for cancer decontamination purposes Only applicable for PTMCs
Magnetic-activated cell sorting (MACS)	95% GC purity 40-fold SSC-activity 0.9–4.6% leukemic cells remaining in the sorted fraction [30]	Inefficient cancer cell decontamination Lack of optimal markers for 100% testicular cell selection Risk of magnetic-bead contamination requiring magnetic-bead removal steps

**Table 2:** Overview of queries used for the systematic review.

Queries
Leydig cell AND isolation
Leydig cell AND marker
Macrophage AND isolation AND testis
Macrophage AND marker AND testis
Peritubular cell AND isolation
Peritubular cell AND marker
Sertoli cell AND isolation
Sertoli cell AND marker
Spermatogonia AND isolation
Spermatogonia AND marker
Spermatogonial stem cell AND isolation
Spermatogonial stem cell AND marker
Testicular cell AND isolation

## Data Availability

Data are contained within the article.

## Abbreviations

The following abbreviations are used in this manuscript:

3β-HSD	3β-Hydroxysteroid Dehydrogenase
α-SMA	Alpha Smooth Muscle Actin
ABP	Androgen binding protein
AR	Androgen receptor
ACTA2	Actin alpha 2
BAX	BCL2 associated X
BMP4	Bone morphogenetic protein 4
CYP11A1	Cytochrome P450 family 11 subfamily A member 1
DAZL	Deleted in Azoospermia-like
DMC1	DNA meiotic recombinase 1
DMEM	Dulbecco’s modified eagle medium
DPG	Discontinuous Percoll gradient
DPPA5	Developmental pluripotency associated 5
DSA	Datura Stramonium Agglutinin
ECM	Extracellular Matrix
EDTA	Ethylenediaminetetraacetic acid
EGF	Epidermal growth factor
EPCAM	Epithelial Cell Adhesion Molecule
FACS	Fluorescent-Activated Cell Sorting
FGF2	Fibroblast growth factor 2
FGFR3	Fibroblast Growth Factor Receptor 3
FSH	Follicle stimulating hormone
GATA-1	GATA Binding Protein 1
GATA-4	GATA Binding Protein 4
GC	Germ Cell
GDNF	Glial cell line-derived neurotrophic factor
GFRA	Growth Factor Receptor A
GPR125	G-Protein Coupled Receptor 125
HBSS	Hanks’ balanced salt solution
HLA	Human Leukocyte Antigen
HNP	Human nuclear protein
hTERT	Human telomerase reverse transcriptase
ICC	Immunocytochemistry
ITGA6	Integrin Subunit Alpha 6
ITGA9	Integrin Subunit Alpha 9
ITT	Immature Testicular Tissue
IU	International unit
IVF-ICSI	In Vitro Fertilization–Intracytoplasmatic Sperm Injection
IVM	In Vitro Maturation
KU	Kunitz unit
LC	Leydig Cell
LHR	Luteinizing Hormone Receptor
MACS	Magnetic-Activated Cell Sorting
MAGEA4	Melanoma-associated antigen 4
MRD-PCR	Minimal Residual Disease Polymerase Chain Reaction
NA	Not available
NGFR	Nerve Growth Factor Receptor
NOA	Non-obstructive azoospermia
OA	Obstructive azoospermia
OCT4	Octamer-Binding Transcription Factor 4
PCNA	Proliferating cell nuclear antigen
PDGFR-α	Platelet-Derived Growth Factor Receptor Alpha
PIWIL2	Piwi-like RNA-mediated gene silencing 2
PLPPR3	Phospholipid Phosphatase Related 3
PLZF	Promyelocytic leukemia zinc finger
PRM2	Protamine 2
PTMC	Peritubular Myoid Cell
RCF	Relative centrifugal force
RPM	Revolutions per minute
RT-PCR	Reverse Transcription Polymerase Chain Reaction
SALL4	Sal-like protein 4
SC	Sertoli Cell
SCF	Stem cell factor
SCP3	Synaptonemal complex protein 3
sLC	Stem Leydig Cell
SOX9	SRY-box 9
SSC	Spermatogonial Stem Cell
SSEA-4	Stage-Specific Embryonic Antigen-4
StAR	Steroidogenic Acute Regulatory Protein
STRA8	Stimulated By Retinoic Acid 8
TESE	Testicular sperm extraction
THY1	Thymocyte Antigen 1
UTF1	Undifferentiated Embryonic Cell Transcription Factor 1
VIM	Vimentin
WT-1	Wilms’ tumor gene 1
ZBTB16	Zinc finger and BTB domain-containing protein 16
